# Chronic Immune Activation in HIV-1 Infection Contributes to Reduced Interferon Alpha Production via Enhanced CD40:CD40 Ligand Interaction

**DOI:** 10.1371/journal.pone.0033925

**Published:** 2012-03-21

**Authors:** Norbert Donhauser, Kathrin Pritschet, Martin Helm, Thomas Harrer, Philipp Schuster, Moritz Ries, Georg Bischof, Jörg Vollmer, Sigrun Smola, Barbara Schmidt, for the German Competence Network HIV/AIDS

**Affiliations:** 1 Institute of Clinical and Molecular Virology, German National Reference Centre for Retroviruses, Friedrich-Alexander-Universität Erlangen-Nürnberg, Erlangen, Germany; 2 Praxis Dr. G. Abelein/Dr. M. Helm, Nürnberg, Germany; 3 Department for Internal Medicine III, University Hospital Erlangen, Friedrich-Alexander-Universität Erlangen-Nürnberg, Erlangen, Germany; 4 Pfizer Oligonucleotide Therapeutics Unit, Coley Pharmaceutical GmbH, Düsseldorf, Germany; 5 Institute of Virology, Saarland University, Homburg/Saar, Germany; University Hospital Zurich, Switzerland

## Abstract

Although a signature of increased interferon (IFN-)alpha production is observed in HIV-1 infection, the response of circulating plasmacytoid dendritic cells (PDC) to Toll-like receptor ligand stimulation is substantially impaired. This functional PDC deficit, which we specifically observed in HIV-1 infected individuals with less than 500 CD4+ T cells/µl, is not well understood. We provide evidence that the peripheral IFN-alpha production in HIV-1 infection is actively suppressed by the enhanced interaction of CD40 ligand (CD40L), a member of the tumor necrosis factor family, and its receptor CD40, which are both upregulated upon immune activation. Plasma levels of soluble CD40L were significantly higher in untreated HIV-1 infected individuals (n = 52) than in subjects on long-term antiretroviral therapy (n = 62, p<0.03) and in uninfected control donors (n = 16, p<0.001). Concomitantly, cell-associated CD40L and the expression of the receptor CD40 on the PDC were significantly upregulated in HIV-1 infection (p<0.05). Soluble and cell-associated CD40L inhibited the PDC-derived IFN-alpha production by CpG oligodeoxynucleotides dose-dependently. This suppressive effect was observed at much lower, physiological CD40L concentrations in peripheral blood mononuclear cells (PBMC) of HIV-1 infected individuals compared to controls (p<0.05). The CpG-induced IFN-alpha production in PBMC of HIV-1 infected donors was directly correlated with PDC and CD4+ T cell counts, and inversely correlated with the viral loads (p<0.001). In HIV-1 infected donors with less than 500 CD4+ T cells/µl, the CpG-induced IFN-alpha production was significantly correlated with the percentage of CD40-expressing PDC and the level of CD40 expression on these cells (p<0.05), whereas CD40L plasma levels played a minor role. In addition, low-dose CD40L contributed to the enhanced production of interleukin 6 and 8 in PBMC of HIV-1 infected donors compared to controls. Our data support the conclusion that the chronic immune activation in HIV-1 infection impairs peripheral PDC innate immune responses at least in part via enhanced CD40:CD40L interactions.

## Introduction

Evidence is accumulating from human and simian studies that chronic immune activation with enhanced T-cell turnover and apoptosis plays a crucial role in lentiviral pathogenesis [1]. An important trigger of immune activation are the type I interferons (IFN), mainly produced by plasmacytoid dendritic cells (PDC) [2], [3]. PDC express Toll-like receptors (TLR) 7 and 9 for the recognition of single-stranded RNA and CpG-like DNA, respectively. Upon stimulation, proinflammatory cytokines are secreted that initiate early immune responses. High-titered HIV-1 and in particular HIV-1 infected cells induce major IFN-alpha production [4]–[6]. The antiviral activity, however, is counteracted by the apoptosis of uninfected CD4+ bystander cells via enhanced expression of the tumor necrosis factor (TNF)-related apoptosis-inducing ligand and its death receptor 5 [7].

The signature of increased expression of IFN-stimulated genes in peripheral cells and lymphatic tissue [8], [9] faces an impaired IFN-alpha production upon TLR stimulation in circulating mononuclear cells and PDC of HIV-1 infected individuals [10]–[14]. This reduced responsiveness to stimulation was recently associated with prior activation of PDC via type I IFNs or virions *in vivo* [15]. Another cause may be a cellular factor induced upon immune activation. In this respect, we focused on the costimulatory molecule CD40 and its ligand, CD40L (CD154), because we and others have shown that CD40L reduced the IFN-alpha production at high concentrations [5], [16]. CD40 was identified on B cells, monocytes, dendritic cells, endothelial and epithelial cells [17]. CD40L, a member of the TNF family, is primarily expressed on activated CD4+ T cells and on a small proportion of CD8+ T cells and platelets [17]. The interaction of CD40 and CD40L is important for activating antigen presenting cells, T cells and macrophages, antibody isotype switching, and germinal center formation. CD40L may be expressed as a heteromultimeric complex [18]. After cleavage of the transmembrane protein by naturally occurring metalloproteinases, the soluble form (sCD40L) still binds to CD40 and delivers biological signals like a cytokine [19].

In HIV-1 infection, CD40 is upregulated on myeloid and plasmacytoid dendritic cells in the peripheral blood [20] and lymphoid tissue [21], [22]. Increased sCD40L levels are detected in the serum of HIV-1 infected subjects and cerebrospinal fluid of patients with AIDS dementia [23], [24]. Controversial data are reported for cell-associated CD40L (cCD40L). Enhanced expression at baseline was observed on CD4+ cells of HIV-1 infected patients, reversed by antiretroviral therapy [25], and on platelets [26]. In contrast, others reported comparable cCD40L levels on resting CD4+ T cells in HIV-1 infected children and uninfected controls [27]. Upon stimulation, CD40L was not sufficiently upregulated on CD4+ cells of HIV-1 infected subjects, which may result in deficient activation of dendritic cells and an ineffective cellular immune response [28]–[30].

In our study, we hypothesized that the HIV-1 induced immune stimulation is directly linked with the decreased PDC IFN-alpha production upon stimulation. We focused on the role of CD40L and provide evidence that physiological concentrations of this molecule reduce the TLR-induced IFN-alpha production in HIV-1 infection.

## Materials and Methods

### Ethics statement

All protocols for this study were approved by the ethical committee of the Medical Faculty, Friedrich-Alexander-Universität Erlangen-Nürnberg (Ref. no. 3375). Ethical guidelines according to the declaration of Helsinki were adhered to, and written informed consent was obtained from the study participants.

### Patient characteristics

**Figure 1:**
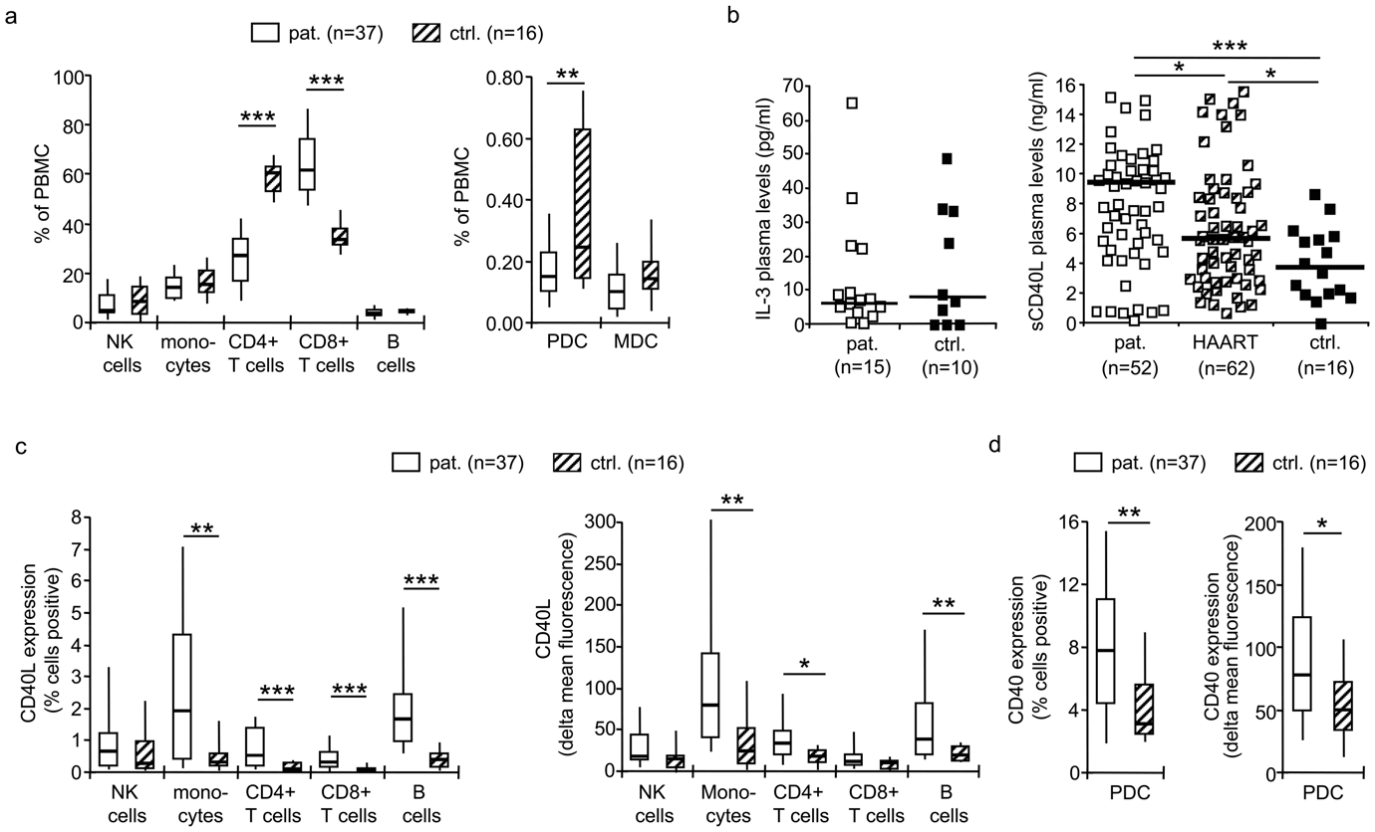
Levels of soluble and cell-associated CD40 ligand in HIV-1 infected patients (pat.) and controls (ctrl.). (**a**) Baseline counts of natural killer (NK) cells, monocytes, CD4+ and CD8+ T cells, B cells, plasmacytoid (PDC) and myeloid dendritic cells (MDC) in HIV-1 infected individuals and uninfected controls, given as percentages (%) of peripheral blood mononuclear cells (PBMC). (**b**) Plasma levels of interleukin (IL-)3 and soluble CD40 ligand (sCD40L), the latter also analyzed in patients on antiretroviral therapy (HAART). (**c**) Expression of cell-associated CD40L (cCD40L) and (**d**) CD40 at the cell surface, evaluated by flow cytometry as percentage of positive cells and delta mean fluorescence intensity after subtraction of isotype controls. Data are presented as median, interquartile ranges (boxes), and 10% and 90% values (whiskers). *p<0.05, **p<0.01, ***p<0.001 (Mann-Whitney test).

The HIV-1 infected subjects (n = 44) were monitored at two specialized outpatient centers in Nürnberg and Erlangen, Germany. None of them had received antiretroviral therapy. Controls (n = 16) were age-matched healthy volunteers at the Institute of Virology, Erlangen, Germany (patients vs. controls, median 41.3 vs. 41.1 years, range 25–68 vs. 25–67 years, p = 0.73, n.s.). Data of some participants were reported recently [31]. Details on baseline cell counts of HIV-1 infected patients and controls are given in **Fig. 1a**. Since the study developed over time, not all values were obtained from the beginning and could not be repeated later on, because antiretroviral therapy was initiated. However, all values that were obtained are reported.

### Isolation and stimulation of cells

EDTA-anticoagulated blood was processed within a few hours (h). PBMC were isolated using Ficoll (Biochrom AG, Berlin, Germany) density gradient centrifugation, and plated at 10^6^ cells/500 µl in 24-well flat bottom plates. Viability of cells was checked by trypan blue staining. PBMC were cultivated in RPMI 1640 media (Gibco, Eggenstein, Germany) plus 10% heat-inactivated fetal calf serum (FCS) (Lonza, Basel, Suisse), 50 mg/ml glutamine, 200 U/ml penicillin, and 90 U/ml streptomycin. IL-3 (R&D Systems, Wiesbaden-Nordenstadt, Germany) was added at indicated concentrations. Cells were stimulated directly after isolation. Supernatants were harvested and stored at −20°C. For the induction of CD40L, cells were stimulated with 1 µg/ml ionomycin und 10 ng/ml PMA (both Sigma-Aldrich) [32]. CD4+ and CD8+ T cells as well as PDC were purified from PBMC by positive selection (Miltenyi Biotec, Bergisch-Gladbach, Germany).

### Determination of cytokines

Cell culture supernatants were analyzed using an IFN-alpha 2a/2b ELISA module set (Bender MedSystems, Vienna, Austria). Samples were measured within the linear range of the assay. IL-3 and sCD40L concentrations were determined using the Quantikine Immunoassay human IL-3 (R&D Systems) and the human sCD40L module set (Bender Medsystems), respectively. Other cytokines were measured using the FlowCytomix technology (Bender MedSystems).

### Generation of the viral stock

The X4-tropic primary virus isolate HIV-1_SF33_ was grown on PHA-stimulated CD4+ T cells. At peak viral replication, supernatants were transferred to PHA-stimulated PBMC. Supernatants were filtered through 0.22 µm filter devices (Millipore, Schwalbach, Germany), aliquoted and stored at −80°C. The TCID_50_ was quantified in PHA- and IL-2-stimulated PBMC [33].

### Cell lines

CD40L-expressing BHK and control cells [34] were grown in Dulbecco's modified Eagles medium (DMEM) (Gibco), supplemented with 10% FCS and antibiotics (see above). For the production of sCD40L-containing or control supernatants, BHK cells were grown to subconfluency, washed twice with DPBS, and fed with serum-free DMEM. After 24 h, supernatants were harvested and concentrated 100-fold using the Centricon Plus-70-10 Centrifugal Filter devices (Millipore). Aliquots were stored at −80°C. To determine the effects of cCD40L, BHK cells were plated at different concentrations into 24-well flat bottom plates. The next day, the plates were incubated on ice for a few minutes. Cells were harvested, washed and fixed in 1.5 ml cryotubes (Eppendorf, Hamburg, Germany) using 4% PFA in DPBS, washed again six times in DPBS, and then added to the PBMC.

### Biologic reagents

Cell-culture grade sCD40L and the respective CD40L antibody were obtained from R&D Systems. Endotoxin-free CpG-A (ODN 6016, 5′-T*C-G-A-C-G-T-C-G-T-G-G*G*G*G-3′, *indicates phosphorothioate and – phosphodiester bonds) and CpG-P (ODN 21798, 5′-T*C-G*T*C-G*A*C-G*A*T*C-G*G*C*G*C-G*C*G*C*C*G-3′) were provided by the Pfizer Oligonucleotide Therapeutics Unit – Coley Pharmaceutical GmbH (Düsseldorf, Germany) and used at 0.75 µM and 0.25 µM, respectively. The synthetic TLR7 agonist S-27609, kindly provided by Richard Miller, 3M Pharmaceuticals (St. Paul, UK), was used at 5 µM.

### Western Blotting

The SDS-denatured sCD40L preparations were separated on a 10% polyacrylamide gel, transferred to a PVDF membrane, incubated with anti-CD40L over night, and then a secondary horseradish peroxidase-conjugated antibody (Dako, Hamburg, Germany; 1∶1,000). The protein size was assessed using the Page Ruler Prestained Protein Ladder (Fermentas, St. Leon-Rot, Germany). Luminescence was developed by adding ECL solution containing luminol (Sigma-Aldrich, Taufkirchen, Germany), documented using the Fujifilm LAS-1000 plus gel documentation system.

### FACS analysis

Isolated PBMC were washed with DPBS supplemented with 1% FCS and 0.5 mM EDTA (Sigma-Aldrich). FcR-blocking reagent (Miltenyi Biotec) was added at 4°C for 10 minutes (min) to reduce unspecific staining. PDC and myeloid dendritic cells were stained using a cocktail of PE-conjugated lineage antibodies (CD3, CD14, CD16, and CD20; Immunotools, Friesoythe, Germany), PE-Cy5-conjugated anti-CD11c, and PE-Cy7-conjugated anti-CD4 (both BD Bioscience, Heidelberg, Germany). B cells were identified using PE-Cy5 conjugated anti-CD19 and APC-conjugated anti-CD20 (Immunotools). CD4+ and CD8+ T-lymphocytes, monocytes, and natural killer (NK) cells were stained using a cocktail of PE-conjugated anti-CD16 (BD Bioscience), PE-Cy5-conjugated anti-CD14 (Immunotools), Pacific Blue-conjugated anti-CD3, PE-Cy7-conjugated anti-CD4 (BD Bioscience) and APC-conjugated anti-CD8 (Immunotools). CD40 and CD40L (CD154) were stained using FITC-conjugated antibodies (eBioscience, Frankfurt, Germany). CD4+ T cell memory subsets were identified using CD3-PB and CD4-PE-Cy7 (both BD Bioscience) in addition to CD45RO-APC and CD197(CCR7)-PE (both eBioscience). Respective mouse IgG antibodies served as isotype controls. After staining at 4°C for 20 min, cells were washed and fixed in 4% paraformaldehyde (Sigma-Aldrich, Taufkirchen). Depending on the expected cell frequency, a total of 100,000–400,000 events were collected using a four-color FACS Calibur with CellQuest 3.3 software (BD Biosciences). CD4+ T cell memory subsets were dissected using an LSR II. The data were analyzed using FCS Express V3.

### Statistics

The Mann-Whitney U-test was used for comparisons between patients and controls. Independently repeated experiments were compared using the paired and unpaired Student's t-test. All statistical calculations assumed a two-sided significance at p values < 0.05.

## Results

### Patient characteristics

We studied 52 untreated HIV-1 infected subjects (48 male, 4 female). All males except two were infected through sex with men. The participants were classified into CDC categories A1 (n = 16), A2 (n = 20), A3 (n = 2), B1 (n = 4), B2 (n = 6), B3 (n = 1), C2 (n = 1), and C3 (n = 2). AIDS-defining illnesses were pneumocystis pneumonia (n = 1), chronic diarrhea and wasting (n = 1), candida oesophagitis (n = 1), and HIV-1 associated encephalopathy (n = 2). Eight patients suffered from chronic bronchitis and/or allergic coryza, four from lues, two from chronic hepatitis, and one each from proctitis, anal fistula, sigma diverticulitis, skin mycosis, recurrent herpes genitalis, condylomata accuminata, nephropathia, and pancreatitis. Two patients were diabetic; one hemophiliac, one addicted to illegal drugs, and three addicted to alcohol. The median CD4+ T cell count was 397/µl (interquartile range, IQR, 273–627; range, 19–1780), the median viral load 4.4 log_10_ (IQR, 3.9–5.1 log_10_; range, 1.3–5.8 log_10_). Baseline percentages of the different cell types are included in **Fig. 1a**.

### TLR-ligand induced IFN-alpha production is decreased in HIV-1 infection

**Figure 2:**
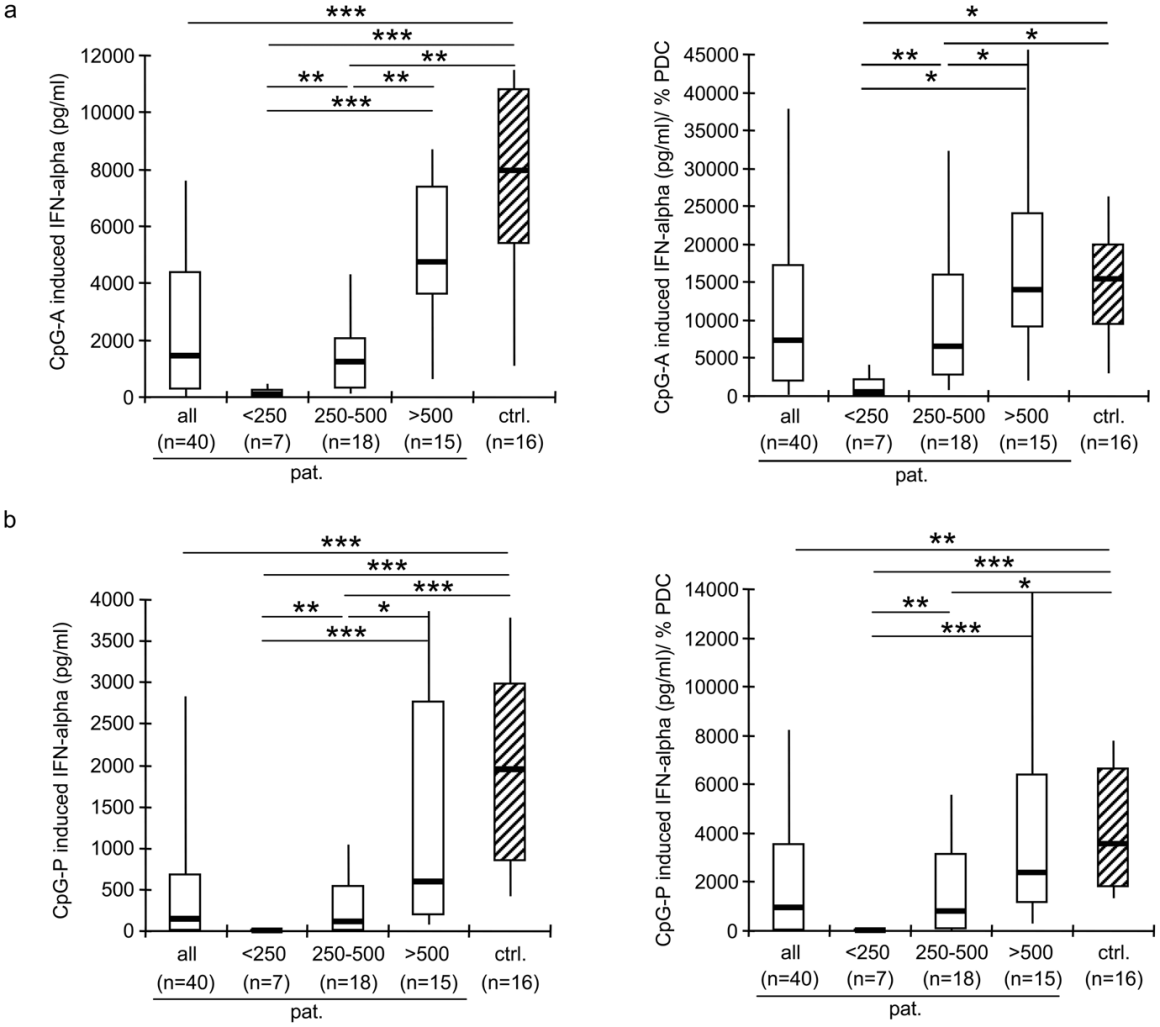
Deficits in interferon (IFN) alpha production in HIV-1 infected patients (pat.) versus control donors (ctrl.). Peripheral blood mononuclear cells (PBMC) were exposed to (**a**) the CpG-A oligonucleotide 6016 (0.75 µM) and (**b**) the CpG-P oligodeoxynucleotide 21798 (0.25 µM) for 20 hours (h). The IFN-alpha production is given before and after normalization for the percentage of plasmacytoid dendritic cells (PDC). The data of HIV-1 infected patients were also analyzed for samples with CD4+ T cell counts below 250/µl (<250), between 250–500/µl (250–500), and above 500/µl (>500). Data are presented as median, interquartile ranges (boxes), and 10% and 90% values (whiskers). *p<0.05, **p<0.01, ***p<0.001 (Mann-Whitney test). Part of the data were presented in another study [31]. Similar data were obtained using S-27609 (data not shown).

The function of PDC was investigated using the oligodeoxynucleotides (ODN) CpG-A (6016) and CpG-P (21798) [35] as TLR9 stimuli (**Fig. 2**). PBMC of HIV-1 infected patients secreted significantly less IFN-alpha than controls upon stimulation with both CpG ODN (p<0.001). When the patients were divided into three groups according to their CD4+ T cell counts, it became obvious that only patients with less than 500 CD4+ T cells/µl contributed to this difference (p<0.01). When the CpG-induced IFN-alpha production was corrected for the percentage of PDC, PBMC of HIV-1 infected patients with CD4+ T cell counts below 500/µl still produced significantly less IFN-alpha compared to controls (p<0.05), whereas for HIV-1 infected patients with more than 500 CD4+ T cells/µl, the normalized IFN-alpha production was not significantly different from control donors. Thus, the functional response of circulating PDC to TLR9 stimulation was substantially impaired in HIV-1 infected subjects with less than 500 CD4+ T cells/µl. The response of PBMC to stimulation with the TLR7 agonist S-27609 was also significantly lower in HIV-1 infected patients compared to controls, before and after correction for the percentage of PDC (both p<0.05; data not shown). In conclusion, both TLR7 and TLR9 responses are impaired in HIV-1 infection.

### sCD40L and cCD40L are significantly elevated in HIV-1 infection

In the search for a cellular factor which is upregulated upon immune activation and suppresses IFN-alpha production, we investigated two molecules: IL-3, secreted by activated CD8+ T cells, which plays an important role in PDC survival and maturation [36]; and CD40L, mainly produced by activated CD4+ T cells, which inhibits the PDC-derived IFN-alpha production at high concentrations [5], [16]. IL-3 plasma levels were similar in HIV-1 infected subjects (n = 15) and controls (n = 10) (median, 6.0 vs. 7.4 pg/ml; IQR 3.4–15.5 pg/ml vs. 1.0–31.0 pg/ml, respectively) (p = 0.89, n.s.) (**Fig. 1b**). The sCD40L plasma levels, however, were significantly elevated in HIV-1 infected patients (n = 52) compared to controls (n = 16) (median, 7.9 vs. 3.7 ng/ml; IQR 5.3–10.3 ng/ml vs. 2.0–5.7 ng/ml, respectively) (p<0.001) (**Fig. 1b**). These data compare to 2-fold increased sCD40L plasma levels in HIV-1 infection reported by others [23]. Similarly, cCD40L was significantly upregulated on monocytes, CD4+ T cells, B cells, and a minority of CD8+ T cells in HIV-1 infected subjects (n = 37) compared to controls (n = 16) (p<0.05) (**Fig. 1c**). Since sCD40L and cCD40L bind to CD40, we studied the expression of CD40 on PDC. As demonstrated by others [20], CD40 was upregulated on the PDC of HIV-1 infected patients (n = 37) compared to controls (n = 16) (p<0.05) (**Fig. 1d**). Notably, ligand and receptor were concomitantly upregulated in HIV-1 infection.

### sCD40L plasma levels are decreased upon antiretroviral treatment

To address the effect of antiretroviral therapy, we analyzed sCD40L plasma levels in a total of 62 HIV-1 infected subjects with undetectable viral loads for at least one year after initiation of antiretroviral therapy. These samples had been sent to the diagnostic services of the Institute of Virology, Erlangen, for routine viral load monitoring. The mean time with viral loads below detection level was 65±23 months (range, 13–95 months). The sCD40L plasma levels were significantly decreased in HAART-treated vs. therapy-naïve patients (p = 0.03), indicating a decrease of immune stimulation with antiretroviral therapy (**Fig. 1b**). However, the sCD40L plasma levels in HAART-treated patients were still significantly higher than in uninfected controls (p = 0.03).

### sCD40L suppresses the PDC-derived IFN-alpha production

**Figure 3:**
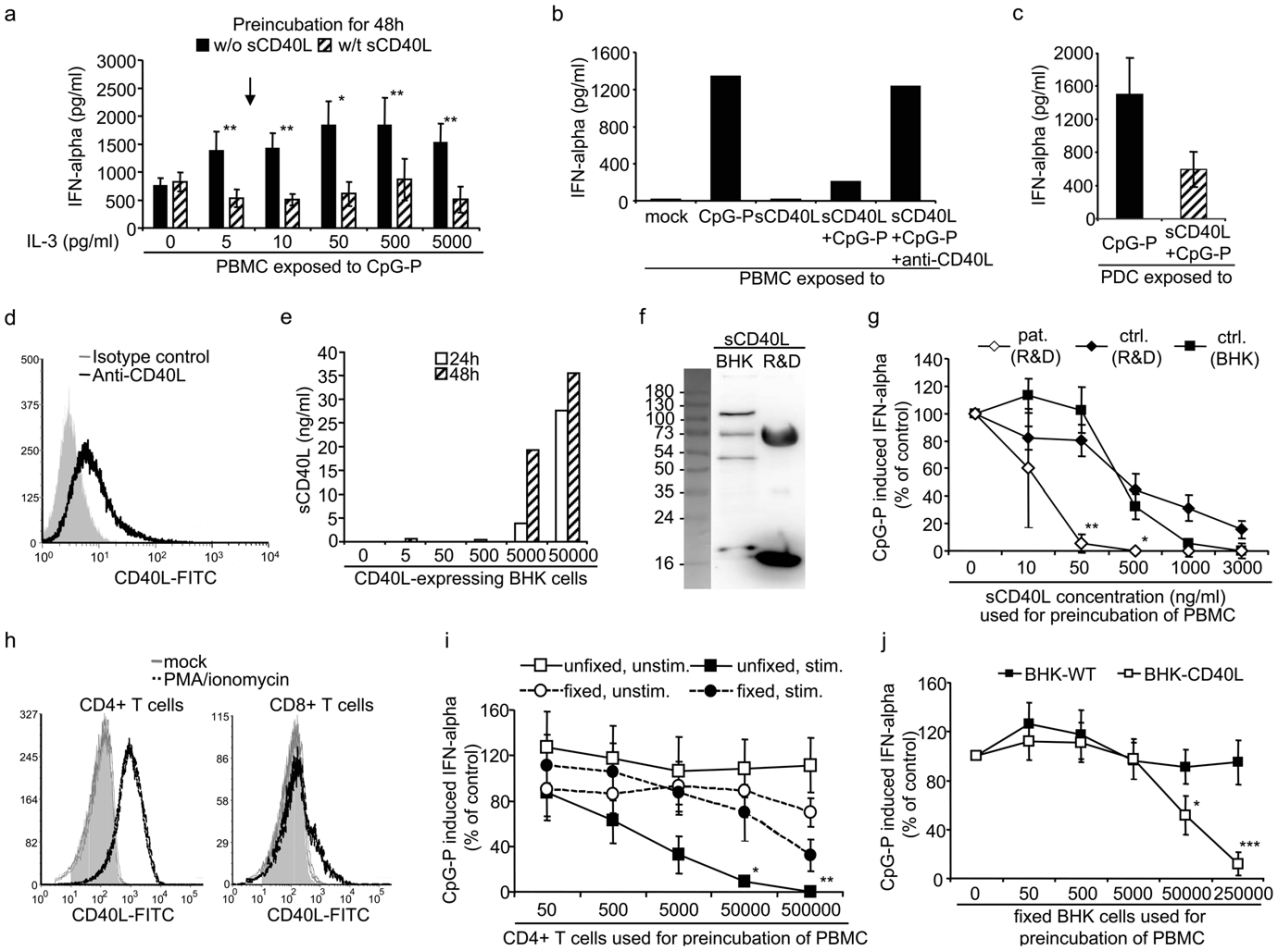
Suppression of interferon (IFN) alpha production by CD40 ligand. (**a**) Preincubation of peripheral blood mononuclear cells (PBMC) of controls without (w/o) or with (w/t) a cell-culture grade commercial soluble CD40 ligand (sCD40L; 3 µg/ml) for 48 hours (h), followed by stimulation with CpG-P 21798. Data represent 11 and 5 separate experiments for IL-3 levels of 0 and 5–5,000 pg/ml, respectively. The arrow indicates physiological IL-3 levels. (**b**) Neutralization of sCD40L with a cell-culture grade anti-CD40L antibody with subsequent CpG-P stimulation in two donors. Similar data were obtained using CpG-A (data not shown). (**c**) Direct effect of sCD40L (3 µg/ml) on the CpG-P induced IFN-alpha production by plasmacytoid dendritic cells (PDC) of control donors. (**d**) Expression of cell-associated CD40L (cCD40L) on baby hamster kidney (BHK) cells. (**e**) Shedding of sCD40L into the cell culture supernatants after 24 h and 48 h of culture. (**f**) Western Blot of concentrated supernatants of CD40L-expressing BHK cells and a commercial sCD40L preparation (R&D Systems) using reducing conditions. Data represent three separate experiments. No bands were detected in the supernatants of BHK wild type cells (data not shown). (**g**) Inhibition of CpG-P induced IFN-alpha production using sCD40L from R&D Systems and BHK cells. Statistics were calculated between PBMC of HIV-1 infected patients (pat.) (n = 5) and controls (ctrl.) (n = 10) exposed to sCD40L (R&D Systems). (**h**) cCD40L expression on CD4+ and CD8+ T cells of control donors after stimulation with mock (grey filled curve) or PMA/ionomycin (black dotted curve). (**i**) Inhibition of CpG-P induced IFN-alpha production by increasing counts of untreated (unstim.) or PMA/ionomycin-stimulated (stim.) CD4+ T cells, which were left unfixed or fixed used 4% paraformaldehyde, and coincubated with 1×10^6^ PBMC of five control donors. Similar results were obtained with CpG-A (data not shown). (**j**) Inhibition of CpG-P induced IFN-alpha production by paraformaldehyde-fixed cCD40L-expressing and wild type (WT) BHK cells using PBMC of eight control donors. Data are presented as mean and standard error. *p<0.05, **p<0.01, ***p<0.001 (Student's t-test).

The effect of sCD40L on the IFN-alpha induction was investigated by preincubation of control PBMC with a cell-culture grade commercial sCD40L (3 µg/ml) for 48 h and subsequent exposure to CpG-P. The IFN-alpha production was significantly reduced by 2–3-fold (p<0.05) at physiological IL-3 levels (5–10 pg/ml) and over a broad range of IL-3 concentrations (5–5,000 pg/ml). (**Fig. 3a**). This did not occur in the absence of IL-3, likely because this cytokine contributes to PDC survival over the three days of incubation [36]. To mimic physiological conditions as close as possible, further experiments were performed using IL-3 concentrations of 5–10 pg/ml. The specificity of the suppressive effect of sCD40L on the IFN-alpha production was proven by neutralization with a respective cell culture-grade antibody (**Fig. 3b**). The inhibitory effect of sCD40L was also observed with purified PDC (**Fig. 3c**), providing evidence for a direct suppressive effect of sCD40L on the PDC-derived IFN-alpha production.

### The effect of sCD40L occurs at physiological levels in HIV-1 infection

The inhibitory effect of the commercial sCD40L was corroborated using a more natural source of this protein. Stably transfected baby hamster kidney (BHK) cells [34] expressed cCD40L at the surface (**Fig. 3d**) and released sCD40L into the cell culture supernatants (**Fig. 3e**). These fluids were concentrated using centrifugal filter devices. sCD40L is a 18 kDa protein, forming trimers [19]. Western blotting showed sCD40L monomers and multimers in the commercial and the BHK-derived preparations (**Fig. 3f**), which were used to titrate the inhibitory effect of sCD40L on the IFN-alpha production in control PBMC. The suppressive effects of both sCD40L preparations were similar with a 50% inhibitory concentration (IC_50_) in the range of 500 ng/ml (**Fig. 3g**). This IC_50_ was considerably above physiological sCD40L levels in control donors (∼50-fold) (**Fig. 1b**). Considering that not only CD40L, but also the receptor CD40 was upregulated on the PDC of HIV-1 infected subjects (**Fig. 1d**), we performed similar experiments using PBMC of these donors. The responsiveness to sCD40L was now substantially increased, the IC_50_ (10 ng/ml) being within sCD40L plasma levels in HIV-1 infected individuals (**Fig. 3g**). Thus, PDC of these subjects in contrast to uninfected donors are susceptible to low physiologic levels of sCD40L.

### Inhibitory effect of cCD40L on the IFN-alpha production

To investigate whether cCD40L also suppressed the IFN-alpha production, CD4+ and CD8+ T cells were stimulated with PMA/ionomycin for 3–4 h. Upon stimulation, cCD40L was upregulated on all CD4+ T cells and on a subset of CD8+ T cells (**Fig. 3h**). In parallel, CD4 was downregulated and CCR7 was upregulated on CD4+ T cells (**Fig. S1**). When CD4+ T cell subsets were compared, central memory CD45RO+ CCR7+ cells upregulated cCD40L comparably to CD45RO− CD4+ T cells, whereas CD45RO+ CCR7− effector memory cells expressed tenfold less cCD40L (**Fig. S1**). Mock- and PMA/ionomycin-stimulated CD4+ T cells were washed, fixed with 4% paraformaldehyde to prevent shedding of CD40L, and coincubated with PBMC of control donors for 2d prior to CpG-P stimulation. The strongest suppressive effects on the IFN-alpha induction were observed with unfixed PMA/ionomycin-stimulated CD4+ T cells (**Fig. 3i**). This effect was seen with as low as 500–5000 CD4+ T cells in 10^6^ PBMC (1∶2000). Minor effects were observed with fixed PMA/ionomycin-stimulated CD4+ T cells, which no longer shed sCD40L. However, these cells still suppressed more than fixed and unfixed unstimulated cells. To reduce the suppressive effect to cCD40L, fixed CD40L-expressing and wild type BHK cells were coincubated with control PBMC. The IFN-alpha production was reduced with high numbers of CD40L-expressing but not wild type BHK cells (**Fig. 3j**), supporting the specific effect of cCD40L in suppressing IFN-alpha production.

### Immune activation suppresses IFN-alpha production at least in part by CD40L

**Figure 4:**
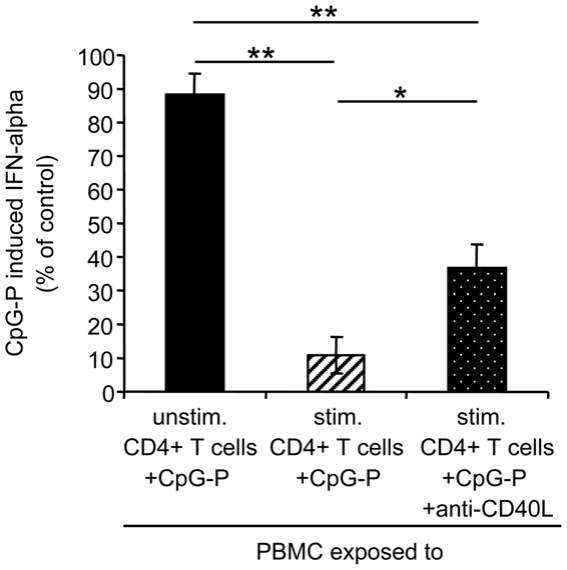
Effect of CD40L neutralization on the suppression of CpG-P induced interferon (IFN)-alpha production. Peripheral blood mononuclear cells (PBMC) of three control donors were preincubated with 50,000 unstimulated (unstim.) or PMA/ionomycin-stimulated (stim.) CD4+ T cells of the respective donors for 48 hours, in the absence or presence of a cell-culture grade anti-CD40L antibody (30 µg/ml), and then stimulated with CpG-P 21798 (0.25 µM) for additional 24 hours. Data are presented as mean and standard error. *p<0.05, **p<0.01 (Tukey HSD test).

So far, we have shown that CD40L suppresses IFN-alpha production. In a next step, we wanted to evaluate the magnitude of the CD40L effect in immune activation. For this purpose, PBMC of three controls donors were preincubated with 50,000 unfixed PMA/ionomycin-stimulated CD4+ T cells with and without the neutralizing CD40L antibody, and then stimulated with CpG-P. The antibody reversed the suppressive effect of PMA/ionomycin-stimulated CD4+ T cells on the IFN-alpha production partially (p<0.05), but not completely (**Fig. 4**). In conclusion, CD40L is an important factor in the immune activation that contributes to the suppression of IFN-alpha production. However, additional factors appear to be involved in this process.

### HIV-1 does not promote shedding of CD40L

To investigate whether HIV-1 infection contributes to shedding of CD40L from the cell surface, PBMC, CD4+ and CD8+ T cells were stimulated with PHA for 3 d, and then infected with HIV-1_SF33_ at a high MOI (0.1) for 2 d. After PMA/ionomycin stimulation, sCD40L was measured in the cell culture supernatants. The majority of sCD40L was detected in the supernatants of CD4+ T cells 4–6 h post stimulation (**Fig. S2**). HIV-1 infection did not contribute to shedding of CD40L under these conditions.

### IFN-alpha production correlates with clinical parameters in HIV-1 infection

**Figure 5:**
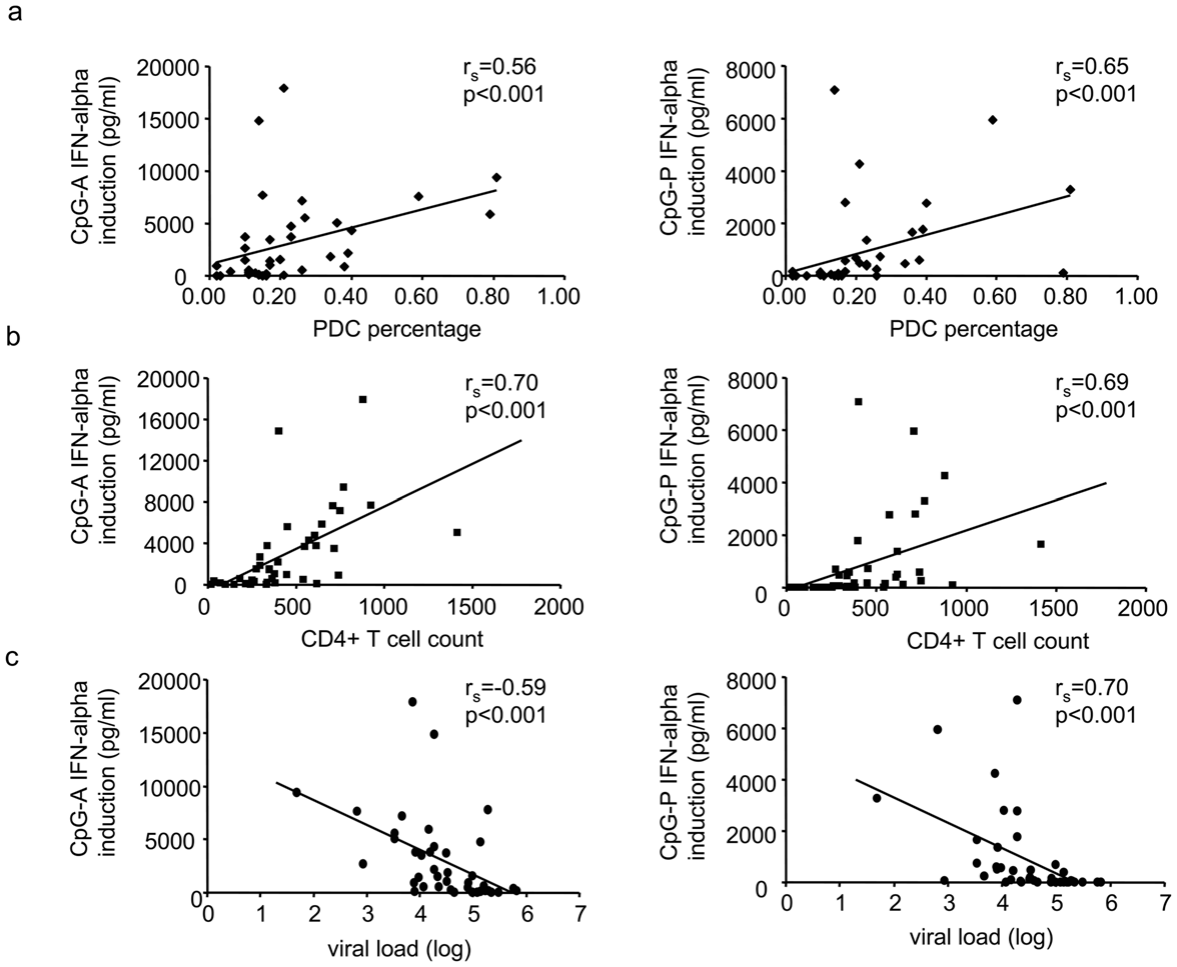
Correlation of CpG-induced interferon (IFN)-alpha production with clinical parameters. Spearman rank order correlation of CpG-A and CpG-P induced IFN-alpha production with (**a**) the percentage of PDC, (**b**) the CD4+ T cell count, and (**c**) the viral load in untreated HIV-1 infected individuals (n = 40).

Next, we studied the influence of PDC and CD4+ T cell counts and viral loads in HIV-1 infected subjects on the CpG-induced IFN-alpha production in PBMC of these donors. The IFN-alpha production was directly correlated with the PDC percentage (**Fig. 5a**) and the CD4+ T cell count (**Fig. 5b**), and inversely correlated with the viral load (**Fig. 5c**) (all p<0.001). Similar data were obtained when the analyses were limited to patients with less than 500 CD4+ T cells/µl (all p<0.001). Thus, all three parameters affect the PDC-derived IFN-alpha production.

### The functional PDC deficit correlates with the expression of CD40 on the PDC in HIV-1 infected subjects with reduced CD4+ T cell counts

Did sCD40L also affect the CpG-induced IFN-alpha production in HIV-1 infected donors? The sCD40L plasma levels were significantly correlated with the CD4+ T cell counts (r_s_ = 0.31, p = 0.03), as similarly reported by others [23], but not with the percentage of PDC (r_s_ = 0.24, p = 0.09, n.s.) nor the viral loads (r_s_ = −0.09, p = 0.54, n.s.). sCD40L plasma levels were also not correlated with the CpG-induced IFN-alpha production, neither for all HIV-1 infected patients nor those with less than 500 CD4+ T cells/µl (p>0.05, n.s.) (**Table 1**). Similar data were obtained when the IFN-alpha induction was normalized for the percentage of PDC (p>0.05, n.s.). Thus, higher sCD40L levels could not explain the functional PDC deficit in HIV-1 infection.

**Table 1:** Correlation of the CpG-induced interferon (IFN) alpha production with sCD40L plasma levels and the expression of CD40 on the PDC in HIV-1 infected subjects.

			HIV-1 infected subjects (n = 40)		HIV-1 infected subjects with <500/µl CD4+ T cells (n = 25)	
Parameter	Stimulus	Readout	r(s)	P	r(s)	P
sCD40L plasma levels (ng/ml)	CpG-A	IFN-alpha (pg/ml)	0.05	0.76 (n.s.)	−0.30	0.15 (n.s.)
		IFN-alpha (pg/ml)/PDC (%)	−0.25	0.12 (n.s.)	−0.25	0.22 (n.s.)
	CpG-P	IFN-alpha (pg/ml)	0.11	0.22 (n.s.)	−0.21	0.32 (n.s.)
		IFN-alpha (pg/ml)/PDC (%)	0.12	0.48 (n.s.)	−0.07	0.72 (n.s.)

			HIV-1 infected subjects (n = 25)		HIV-1 infected subjects with <500/µl CD4+ T cells (n = 15)	
CD40 expression on PDC (MFI)	CpG-A	IFN-alpha (pg/ml)	−0.27	0.21 (n.s.)	−0.59	0.03
	CpG-A	IFN-alpha (pg/ml)/PDC (%)	−0.32	0.13 (n.s.)	−0.36	0.21 (n.s.)
	CpG-P	IFN-alpha (pg/ml)	−0.22	0.31 (n.s.)	−0.70	0.005
	CpG-P	IFN-alpha (pg/ml)/PDC (%)	−0.45	0.03	−0.70	0.005
CD40 expression (% PDC positive)	CpG-A	IFN-alpha (pg/ml)	−0.17	0.42 (n.s.)	−0.58	0.02
	CpG-A	IFN-alpha (pg/ml)/PDC (%)	−0.40	0.05	−0.69	0.004
	CpG-P	IFN-alpha (pg/ml)	−0.13	0.55 (n.s.)	−0.37	0.17 (n.s.)
	CpG-P	IFN-alpha (pg/ml)/PDC (%)	−0.10	0.64 (n.s.)	−0.50	0.05

r(s) = Spearman rank correlation coefficient, MFI mean fluorescence intensity, n.s. not significant.

Next, we addressed the role of the increased expression of CD40 on the PDC in HIV-1 infected individuals. Neither the mean fluorescence intensity nor the percentage of CD40-expressing PDC were correlated with CD4+ T cell counts, PDC percentages, or viral loads, irrespective of whether all HIV-1 infected subjects or those with less than 500 CD4+ T cells/µl were analyzed (p>0.05, n.s.). In contrast, sCD40L plasma levels were significantly correlated with the percentage of CD40-expressing PDC (r_s_ = 0.34, p = 0.04) and the mean fluorescence intensity (r_s_ = 0.40, p = 0.02) in all HIV-1 infected subjects and in patients with less than 500 CD4+ T cells/µl (r_s_ = 0.43, p = 0.04, and r_s_ = 0.56, p = 0.008, respectively). Notably, also the CpG-induced IFN-alpha production was significantly correlated with the expression of CD40 on PDC, when the analysis was limited to patients with less than 500 CD4+ T cells/µl (**Table 1**). In conclusion, enhanced CD40:CD40L interactions play a role for the functional PDC deficit in HIV-1 infected subjects with low CD4+ T cell counts.

### sCD40L modifies the cytokine profile in HIV-1 infection

**Figure 6:**
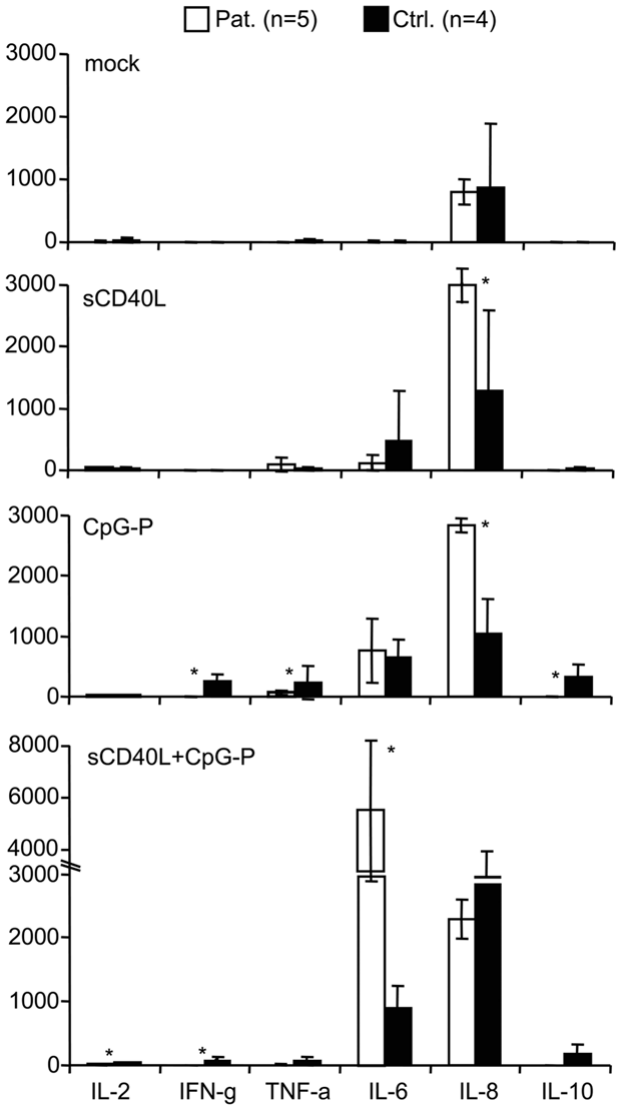
Effect of soluble CD40 ligand (sCD40L) on the cytokine profile. Peripheral blood mononuclear cells (PBMC) of HIV-infected patients (pat.) and control (ctrl.) donors were exposed to low-dose sCD40L (50 pg/ml) for 48 hours and subsequently stimulated with the CpG-P oligodeoxynucleotide 21798 for 20 hours. The levels of interleukin (IL-)2, interferon gamma (IFN-g), tumor necrosis factor alpha (TNF-a), IL-6, IL-8, and IL-10 were measured using a fluorescent bead assay. IL-4, IL-5, IL-12, TNF-beta, and IL-1 beta were not detected. Data are presented as mean and standard error. *p<0.05.

The effects of sCD40L on the cytokine production were determined in PBMC of HIV-infected (n = 5) and control donors (n = 4) using a fluorescent bead immunoassay (**Fig. 6**). A low concentration of sCD40L (50 ng/ml) significantly upregulated IL-8 in the PBMC of HIV-infected subjects (p<0.05). A similar effect was observed after CpG-P stimulation, whereas PBMC of control donors upregulated IFN-gamma, TNF-alpha, and IL-10. Combined stimulation with sCD40L and CpG-P significantly upregulated IL-6 in PBMC of HIV-infected subjects (p<0.05), indicating that low-dose CD40L causes a shift in the cytokine profile.

## Discussion

Our study shows, for the first time, that the upregulation of CD40L and in particular the receptor CD40 on the surface of PDC contributes to reduction of the PDC-derived IFN-alpha production in HIV-1 infection. The sCD40L plasma levels, the expression of cCD40L on several cell populations and the expression of CD40 on the PDC were concomitantly upregulated in HIV-1 infected subjects (**Fig. 1**), which corroborates data by other groups [20]–[24]. *In vitro*, sCD40L obtained from two different sources and cCD40L specifically and dose-dependently reduced the CpG-induced IFN-alpha production (**Fig. 3**). With PBMC of uninfected control donors, the suppressive effect was observed at high sCD40L concentrations only; in contrast, ∼50-fold lower concentrations were sufficient when PBMC of HIV-1 infected subjects were used (**Fig. 3d**). These findings suggest that the concomitant upregulation of CD40L and in particular its receptor on the PDC becomes physiologically relevant. The *in vivo* data support this conclusion; not sCD40L plasma levels, but the enhanced expression of CD40 was significantly correlated with the CpG-induced IFN-alpha production in PBMC of HIV-1 infected donors (**Table 1**). This effect was limited to subjects with less than 500 CD4+ T cells/µl, which remarkably correlated with the fact that the functional PDC deficit was most evident in these donors (**Fig. 2**).

The CpG-induced IFN-alpha production in PBMC of HIV-1 infected subjects was also substantially affected by PDC and CD4+ T cell counts and viral loads (**Fig. 4**), suggesting that the progression of disease negatively impacts PDC functions. PDC and CD4+ T cell counts recover at least partially in subjects on antiretroviral therapy [37], [38]. Therefore, it was interesting to investigate sCD40L plasma levels in HAART-treated patients. Sipsas *et al.* reported that sCD40L plasma levels were correlated with CD4+ T cell counts, and that both markers increased in parallel after 8–12 months of antiretroviral therapy [23]. In contrast, Sousa *et al.* observed a decline of cCD40L expression on CD4+ T cells after eight months of HAART [25], and Barron *et al.* reported a reduced CD40 expression on the PDC of treated HIV-1 infected subjects [20]. We confirmed that sCD40L plasma levels were significantly correlated with the CD4+ T cell counts in our study participants (p = 0.03). In addition, we observed a transient increase of sCD40L plasma levels in most subjects after the initiation of antiretroviral therapy (**Fig. S3**). Notably, this kinetics was delayed in patients with less than 250 CD4+ T cells/µl, most likely reflecting the slower increase of CD4+ T cells in these donors. However, sCD40L plasma levels were significantly lower in patients on long-term antiretroviral treatment compared to untreated subjects (p = 0.03) (**Fig. 1b**), probably reflecting a decrease of immune activation over time. Still, sCD40L plasma levels were higher than in uninfected controls, suggesting that chronic immune activation is not completely resolved by antiretroviral therapy.

The CD40:CD40L interaction is known to be important for activating antigen presenting cells, T cells and macrophages, antibody isotype switching, and germinal center formation [17]. In this respect, the reduction of IFN-alpha production by CD40L may be a physiological reaction, namely to limit the antiproliferative effect of IFN-alpha when antigen-specific CD4+ T cells expand. These adaptive immune responses usually result in the elimination of the pathogen. In HIV-1 infection, however, the virus cannot be eliminated and thus propagates continuous immune activation concomitant with a CD4+ T cell decline. Notably, the suppressive effect of PMA/ionomycin-stimulated CD4+ T cells on the IFN-alpha production was not completely reversed by the neutralizing antibody to CD40L (**Fig. 4**), suggesting that others factors besides CD40L contribute to silencing of PDC innate responses.

Recently, the reduced responsiveness of PDC to stimulation was associated with prior activation of these cells via IFN-alpha or HIV-1 particles *in vivo* [15]. A signature of increased IFN-alpha production has been observed in peripheral cells and in particular in the lymphatic tissue, which obviously contributes to the immunopathogenesis of HIV-1 infection [7]–[9], [39]. How does this reconcile with our data of reduced IFN-alpha production upon enhanced CD40:CD40L interactions? CD40L-expressing CD4+ T cells are present in the periphery and in the lymphatic tissue, corroborated by the enhanced expression of CCR7 of central memory CD4+ T cells upon immune activation (**Fig. S1**), supporting depletion of these cells from the peripheral blood and homing to lymphatic tissue [40]. Importantly, PDC of HIV-1 infected donors can still be activated by strong stimuli, e.g. CpG-ODN or TLR7 agonists, and produce IFN-alpha, although at a lower level than uninfected controls [13], [31], [41]. In this respect, HIV-1 infected cells are a robust stimulus of IFN-alpha production [6], [42], whereas virions are only effective at high titer [4], [5], [7], which we confirmed (**Fig. S4**). Therefore, we propose a model with an increased IFN-alpha production mainly in the lymphatic tissue, induced by cell∶cell contact of PDC with HIV-1 infected CD4+ T cells, whereas the suppressive effect of sCD40L on PDC innate immune responses predominates in the periphery [43]. Along this line, we did not observe increased IFN-alpha levels in the peripheral blood of our study participants (data not shown), which is consistent with a recent report showing comparable IFN-alpha levels in the plasma of aviremic and viremic HIV-1 infected subjects and controls [44]. However, PDC homed to lymphatic tissue should be considered an important source of IFN-alpha. Thus, the imbalanced release of IFN-alpha appears to be crucial for the immunopathogenesis of HIV-1 infection.

The reduced IFN-alpha production in the periphery of HIV-1 infected individuals may compromise the long-term immune control of viral infections and associated tumors, which are in principle susceptible to type I IFN. In this respect, HHV-8-associated Kaposi sarcoma is observed in patients with preserved CD4+ T cell counts; the prevalence of papillomavirus-related anal neoplasia continues to increase in aging HIV-1 infected individuals despite highly active antiretroviral therapy [45]; and the suppressive effect of CD40L may be relevant for the development of EBV-associated Hodgkin lymphoma [46]. It is also notable that genital herpes simplex virus type 2 infections frequently reactivate after the initiation of antiretroviral therapy as hallmark of the immune reconstitution inflammatory syndrome [47], [48]. The flare of opportunistic infections may be provoked by the transient increase of CD40L-expressing CD4+ T cells suppressing type I interferon production. The CD40L effect may be potentiated by the shift in the cytokine profile, namely the reduced secretion of IFN-gamma, TNF-alpha, and IL-10, along with the enhanced production of IL-6 and IL-8 (**Fig. 6**). The latter observation fits to data by others reporting that CD40L is incorporated into viral particles and induces IL-8 production in monocyte-derived macrophages [49].

So far, the mechanism remains unclear how PDC innate immune responses are silenced by CD40L:CD40 interactions, which will be addressed in further studies. Beyond HIV-1 immunopathogenesis, the CD40L effect may be relevant for other infectious diseases associated with chronic immune activation, and autoimmune disorders. It is noteworthy that the latter can present with CD4+ T cell lymphopenia. In this respect, interactions of CD40 and CD40L appear to be a major factor in the immunopathogenesis of chronic immune activation. Thus, treatment of the immune activation without inducing further immunosuppression appears to be a promising goal to reverse the innate immune defect.

## Supporting Information

Figure S1 **Effect of immune stimulation on the expression of CD40 ligand (CD40L) on CD4+ T cell memory subsets.** After stimulation of isolated CD4+ T cells with PMA/ionomycin for 5 h, CD40L expression was analyzed on central memory (CM) and effector memory (EM) cells, which were identified as CD45RO+ CCR7+ and CD45RO+ CCR7− cells, respectively.
(TIF)

Figure S2 **Impact of HIV-1 on the shedding of CD40 ligand (CD40L).** Peripheral blood mononuclear cells (PBMC), CD4+ and CD8+ T cells of five control donors were stimulated with PHA for 3 d, and then infected without (w/o) or with (w/t) HIV-1_SF33_ (MOI 0.1) for 2 d. 1×10^6^ cells each were exposed to PMA/ionomycin, and soluble CD40L (sCD40L) was determined in the cell culture supernatants at indicated time periods. Data are presented as mean and standard error.
(TIF)

Figure S3 **Longitudinal analysis of soluble CD40 ligand (sCD40L) plasma levels in HIV-1 infected patients on highly active antiretroviral therapy (HAART).** All patients of our study with a follow-up of at least six months on antiretroviral therapy (n = 18) were included. For the analysis, patients were divided into two groups with more or less than 250 CD4+ T cells/µl at initiation of HAART.
(TIF)

Figure S4 **Effect of HIV-1 on the induction of interferon (IFN) alpha production.** Peripheral blood mononuclear cells (PBMC) of six control donors were preincubated with infectious or heat-inactivated (95°C, 20 min) HIV-1_SF33_ at decreasing multiplicities of infection (MOI) for 24 hours (h) prior to stimulation with mock or the CpG-P oligodeoxynucleotide 21798 (0.25 µM) for additional 24 h and 48 h (data not shown). Data are presented as mean and standard error.
(TIF)
