# Supplementary figures and images for: Chronic Immune Activation in HIV-1 Infection Contributes to Reduced Interferon Alpha Production via Enhanced CD40:CD40 Ligand Interaction
Source: PLoS One. 2012 Mar 21;7(3):e33925. doi: 10.1371/journal.pone.0033925 (PMC3309969; doi:10.1371/journal.pone.0033925)

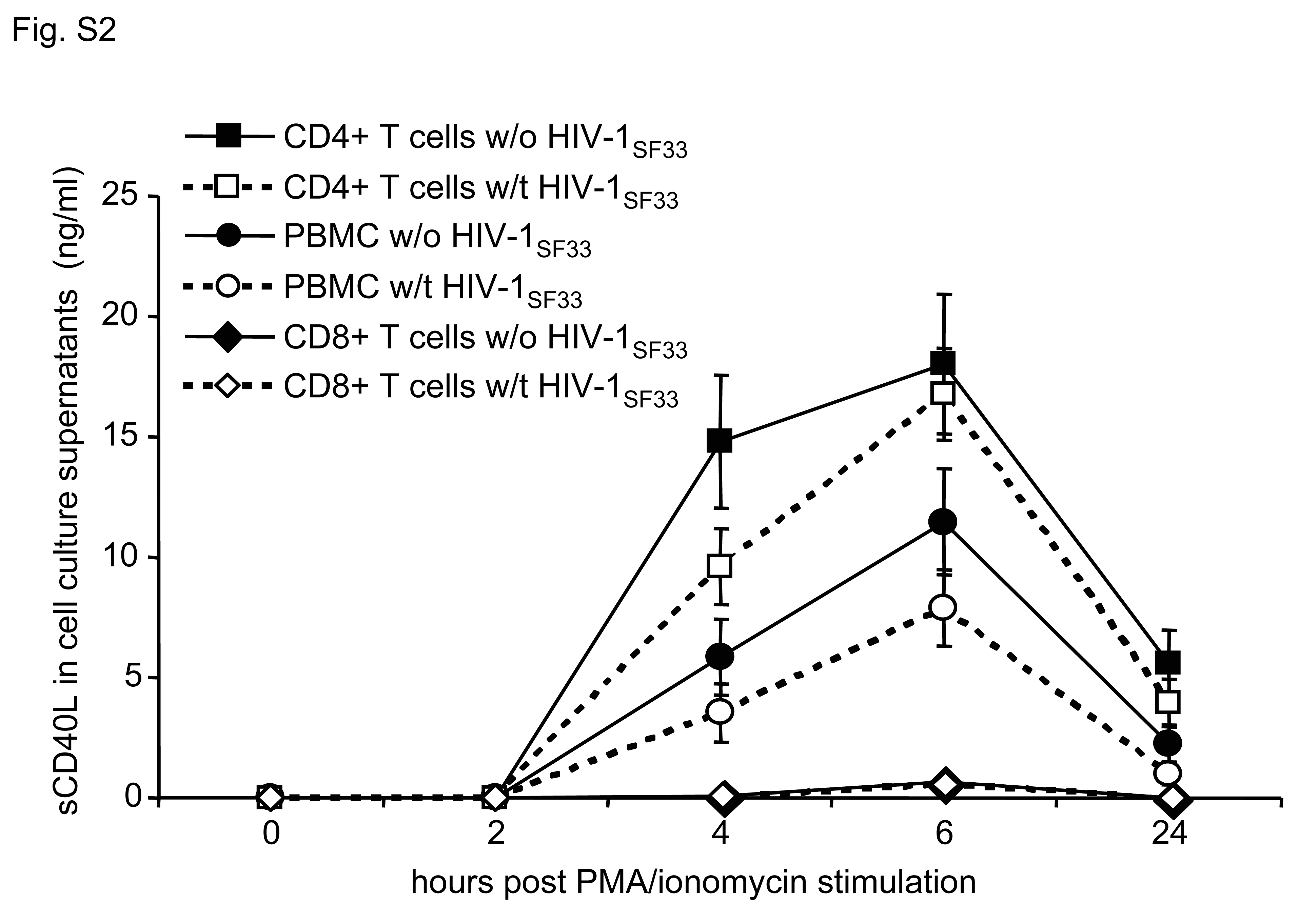

Supplement: Figure S2 — Impact of HIV-1 on the shedding of CD40 ligand (CD40L). Peripheral blood mononuclear cells (PBMC), CD4+ and CD8+ T cells of five control donors were stimulated with PHA for 3 d, and then infected without (w/o) or with (w/t) HIV-1SF33 (MOI 0.1) for 2 d. 1×10^6^ cells each were exposed to PMA/ionomycin, and soluble CD40L (sCD40L) was determined in the cell culture supernatants at indicated time periods. Data are presented as mean and standard error. (TIF) [file pone.0033925.s002.tif]

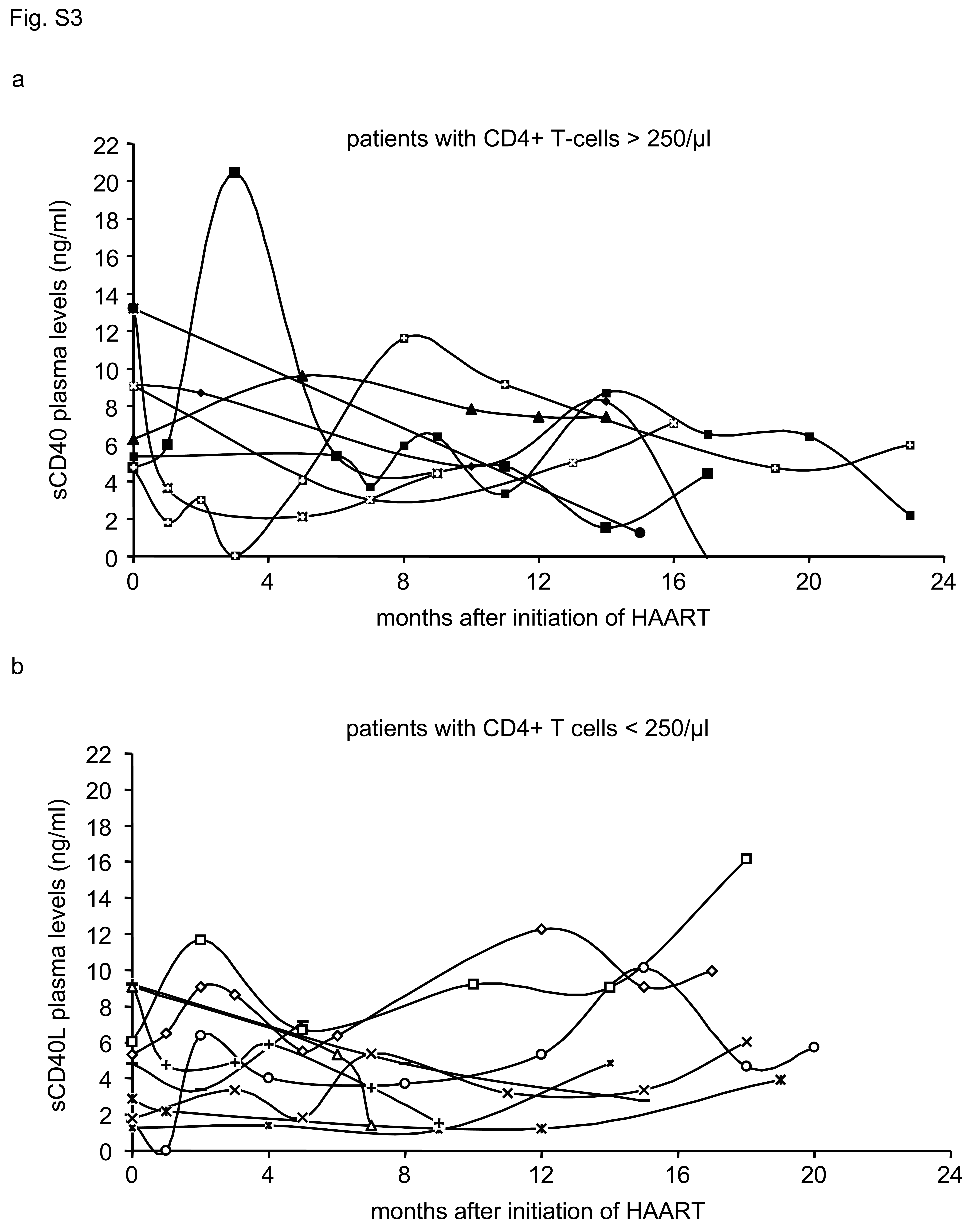

Supplement: Figure S3 — Longitudinal analysis of soluble CD40 ligand (sCD40L) plasma levels in HIV-1 infected patients on highly active antiretroviral therapy (HAART). All patients of our study with a follow-up of at least six months on antiretroviral therapy (n = 18) were included. For the analysis, patients were divided into two groups with more or less than 250 CD4+ T cells/µl at initiation of HAART. (TIF) [file pone.0033925.s003.tif]

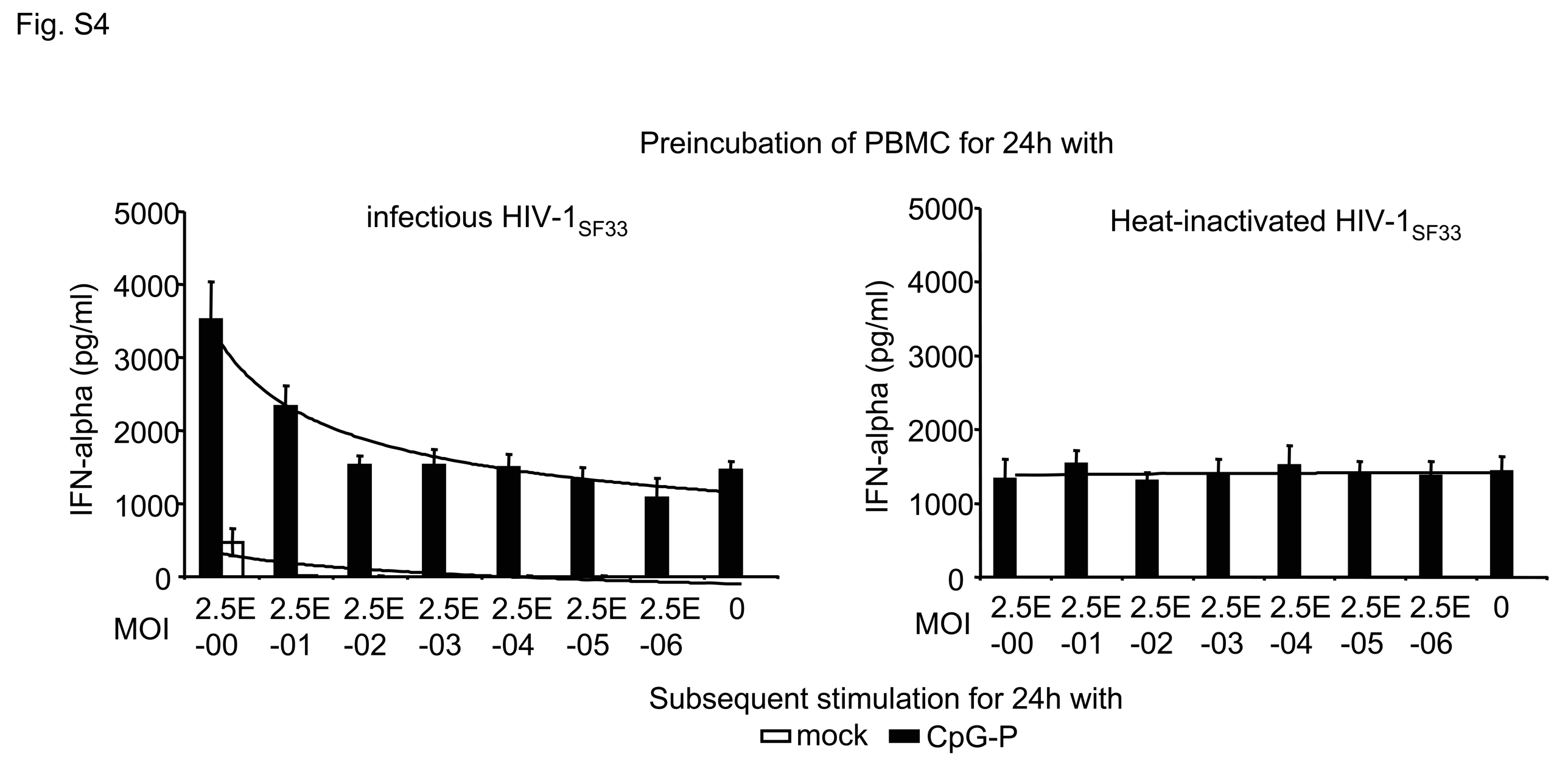

Supplement: Figure S4 — Effect of HIV-1 on the induction of interferon (IFN) alpha production. Peripheral blood mononuclear cells (PBMC) of six control donors were preincubated with infectious or heat-inactivated (95°C, 20 min) HIV-1SF33 at decreasing multiplicities of infection (MOI) for 24 hours (h) prior to stimulation with mock or the CpG-P oligodeoxynucleotide 21798 (0.25 µM) for additional 24 h and 48 h (data not shown). Data are presented as mean and standard error. (TIF) [file pone.0033925.s004.tif]
